# Spatially resolved transcriptomic profiling for glomerular and tubulointerstitial gene expression in C3 glomerulopathy

**DOI:** 10.1093/ckj/sfaf139

**Published:** 2025-05-08

**Authors:** Jung Hun Koh, Minji Kang, Sehoon Park, Ha Yeon Shin, Hyunah Ku, Seong Min Lee, Jeong Min Cho, Semin Cho, Yaerim Kim, Soojin Lee, Hajeong Lee, Kwon-Wook Joo, Kyung Chul Moon, Seung Hee Yang, Hyun Je Kim, Dong Ki Kim

**Affiliations:** Department of Internal Medicine, Seoul National University Hospital, Seoul, Korea; Department of Internal Medicine, Seoul National University College of Medicine, Seoul, Korea; Department of Biomedical Sciences, Seoul National University Graduate School, Seoul, Korea; Department of Internal Medicine, Seoul National University Hospital, Seoul, Korea; Department of Biomedical Sciences, Seoul National University Graduate School, Seoul, Korea; Department of Biomedical Sciences, Seoul National University Graduate School, Seoul, Korea; Department of Biomedical Sciences, Seoul National University Graduate School, Seoul, Korea; Department of Internal Medicine, Seoul National University Hospital, Seoul, Korea; Department of Internal Medicine, Chung-Ang University Gwangmyeong Hospital, Gwangmyeong-si, Gyeonggi-do, Korea; Department of Internal Medicine, Keimyung University School of Medicine, Daegu, Korea; Department of Internal Medicine, Uijeongbu Eulji University Medical Center, Uijeongbu-si, Gyeonggi-do, Korea; Department of Internal Medicine, Seoul National University Hospital, Seoul, Korea; Department of Internal Medicine, Seoul National University College of Medicine, Seoul, Korea; Department of Internal Medicine, Seoul National University Hospital, Seoul, Korea; Department of Internal Medicine, Seoul National University College of Medicine, Seoul, Korea; Department of Pathology, Seoul National University College of Medicine, Seoul, Korea; Kidney Research Institute, Seoul National University, Seoul, Korea; Department of Biomedical Sciences, Seoul National University Graduate School, Seoul, Korea; Department of Internal Medicine, Seoul National University College of Medicine, Seoul, Korea

**Keywords:** C3 glomerulopathy, complement, gene expression, glomerulonephritis, glomerulus

## Abstract

**Background:**

Complement 3 (C3) glomerulopathy (C3G) is a rare but clinically significant glomerulopathy. However, little is known about its transcriptomic profile. We investigated the substructure-specific gene expression profile of C3G using the recently introduced spatial transcriptomics technology.

**Methods:**

We performed spatial transcriptomic profiling using GeoMx Digital Spatial Profiler with formalin-fixed paraffin-embedded kidney biopsy specimens of three C3G cases and seven controls from donor kidney biopsy. Additionally, 41 samples of other glomerulonephritis, including focal segmental glomerulosclerosis, membranous nephropathy and minimal change disease, were included as disease controls. We identified differentially expressed genes (DEGs) specific to C3G, followed by *in vitro* validation analysis of consistently upregulated DEGs in human glomerular endothelial cells through a co-culture with complement-stimulated macrophages.

**Results:**

We found 229 and 157 highly expressed DEGs in the glomeruli of C3G compared with those of donor and disease controls, respectively, including *POSTN, COL1A2* and *IFI44L*. Protease binding, structural molecule activity and extracellular matrix (ECM) structural constituent were among the top enriched Gene Ontology terms in the glomeruli of C3G. Specifically, genes related to the ECM and interferon activity were the most upregulated, with network analysis suggesting possible interactions between complement C3 and the ECM through CD11c. The *in vitro* experimental validation using iC3b-stimulated CD11c+ macrophages supported these findings, inducing elevated expression of fibrosis markers and ECM components in glomerular endothelial cells.

**Conclusions:**

Significant disease-specific transcriptomic alterations in C3G, including upregulation of genes related to the ECM, provide potential insights into the pathophysiology.

## Graphical Abstract

**Graphical Abstract:**
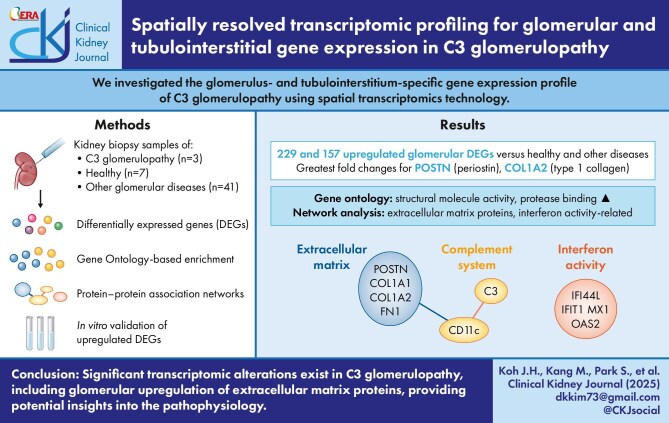

KEY LEARNING POINTS **What was known:**

- Complement 3 (C3) glomerulopathy (C3G) is marked by dysregulation of the alternative complement pathway, although its interactions with the glomerulus have not been fully elucidated.
- Spatial transcriptomics analysis of C3G could reveal the substructure-specific pathways most relevant to the pathophysiology.

**This study adds**:

- C3G was associated with significant glomerulus-specific upregulation of genes related to extracellular matrix (ECM) and interferon activity.
- Network analysis suggested possible interactions between C3 and the ECM in C3G modulated by CD11c, a component of an integrin complement receptor, whose effect on glomerular fibrosis was examined *in vitro*.

**Potential impact:**

- The glomerular ECM, through disease-specific interactions with the complement system, is a potentially important target in further characterizing the pathophysiology of C3G.

## INTRODUCTION

Complement 3 (C3) glomerulopathy (C3G) is a primary glomerulonephritis that results from dysregulation of the complement system [1]. Although it remains a rare disorder, 30%–50% of cases progress to end-stage kidney disease within a decade of diagnosis, with frequent recurrence after kidney transplantation [2–4]. Medical management is currently limited to supportive care along with selective use of immunosuppressive agents and plasmapheresis, with mixed reports on their efficacy [5]. While complement pathway inhibitors have been proposed as potential disease-specific therapies, recent studies of the C5 inhibitor eculizumab in C3G have not shown consistent efficacy [6–8].

The recent recognition of C3G as a distinct histopathological entity has allowed more precise characterizations of the underlying pathophysiology. Studies based on familial cases and animal models have shown that constitutive activation of the alternative pathway is key to the development of the disease, mediated by factors such as mutations in complement regulator genes and autoantibodies to enzymes in the complement cascade [1, 9]. At the same time, components of the glomerular microenvironment, which include the glomerular glycocalyx and epithelial cells, interact with complement regulators and appear to play a crucial role in local regulation of the complement pathway [1, 10]. Given the structural complexity of the glomerular microenvironment, however, how the relevant molecular pathways integrate and manifest as C3G remains to be elucidated.

One evolving approach in addressing such questions in kidney diseases has been to employ spatial transcriptomics [11]. Spatial transcriptomics technologies enable mapping of gene expression profiles to specific histological structures, which may be as small as individual glomeruli [11, 12]. Spatial transcriptomics profiling of the kidney in C3G may reveal patterns of gene expression that indicate the most relevant molecular pathways and networks in the pathophysiology of the disease.

In the present study, we employed the spatial transcriptomics framework to analyze gene expression patterns in the glomeruli and tubules of C3G. We hypothesized that C3G would have distinct substructure-specific transcriptomic changes in comparison with healthy donor kidneys and other common glomerular diseases. Comparison with healthy donor kidneys would be more sensitive in detecting all transcriptomic changes involved in C3G, while comparison with other glomerular diseases with distinct pathologic mechanisms would reveal specific changes in local expressions of genes that are unique to the complement-mediated pathogenesis of C3G.

## MATERIALS AND METHODS

### Ethical considerations

This study was approved by the Institutional Review Board of Seoul National University Hospital (IRB No. 2208-137-1353). Informed consent was waived by the IRB due to the retrospective nature of the study based on archived formalin-fixed paraffin-embedded samples. The study was conducted in accordance with the Declaration of Helsinki.

### Sample selection and collection

Study samples were selected from kidney biopsies performed at Seoul National University Hospital between 2009 and 2021 with a standardized protocol for fixation and embedding. Disease controls included minimal change disease (MCD), membranous nephropathy (MN), focal segmental glomerulosclerosis (FSGS) and diabetic nephropathy (DN). Time-zero allograft biopsies from living donor kidney transplantation were used as healthy controls. The inclusion criteria were set as follows: (i) age ≥18 years, (ii) ≥10 glomeruli per section, (iii) estimated glomerular filtration rate (eGFR) ≥30 mL/min/1.73 m^2^, and (iv) absence of histopathologic features of DN in non-DN cases. Donor controls were further specified to have eGFR ≥80 mL/min/1.73 m^2^ and be pathologically free of kidney disease.

### Slide preparation and processing

Kidney tissues were mounted, followed by deparaffinization and antigen retrieval. *In situ* hybridization was performed with the GeoMx Whole Transcriptome Atlas, which consists of RNA probes conjugated with unique ultraviolet-photocleavable oligonucleotide barcodes targeting 18 677 genes. The slides were then stained with morphology markers SYTO 13, Pan-cytokeratin and alpha-smooth muscle actin.

The slides were loaded into the GeoMx Digital Spatial Profiler (DSP) instrument, and representative glomerular and tubulointerstitial substructures were selected as regions of interest (ROIs) by a kidney pathologist. The oligonucleotide barcodes for the target genes within each ROI were photocleaved and collected into a DSP collection plate. The oligonucleotides underwent amplification, purification and quality control. Finally, they were sequenced on an Illumina NovaSeq 6000. The full preparation and processing protocol is described in the Supplementary Methods.

### Bioinformatics and statistical analysis

Raw sequencing reads in FASTQ format were converted into digital count conversion files using the GeoMx NGS Pipeline (v2.0). The DSP Data Analysis Suite (v2.4) was used for data analysis and quality control. For each gene, low performance probes whose average read counts fell below 10% of the average across all probes were excluded, as were outlier probes detected by applying the Grubbs test to all probes in each ROI. The limit of quantitation for gene expression was set as 2.0 geometric standard deviations above the geometric mean of negative probes. Genes expressed in <50% of the collected ROIs were filtered out, and substructure-specific gene expression levels were checked to confirm specificity of the selected ROIs. To identify differentially expressed genes (DEGs) between C3G and controls, we used the DESeq2 package in R (version 3.6.2), which utilizes negative binomial generalized linear models [13]. The model was specified to use median-of-ratios method for normalization, parametric fitting for dispersion estimation and Wald tests for statistical significance evaluation. DEGs with a false-discovery rate <0.05 by the Benjamini–Hochberg correction were considered significant. The DEGs were analyzed using the ToppGene Suite for functional enrichment analysis based on Gene Ontology annotations [14]. To investigate the protein-protein interaction networks among the DEGs, we used the STRING database [15]. For each association, STRING assigns a confidence score between 0 and 1 corresponding to the likelihood of a true functional interaction. The DEGs were mapped based on confidence scores at a 0.7 threshold, and DEGs with at least one significant interaction were presented.

### *In vitro* validation experiments

The expressions of periostin, Col1a1 and fibronectin in samples included in the transcriptomic profiling were assessed with immunohistochemistry. Next, macrophages were isolated from THP-1 monocyte cell line, and CD11c+ macrophages were selected with flow cytometry. Human glomerular endothelial cells (hGECs) were sourced from nephrectomy specimens from renal cell carcinoma patients, as described previously [16]. CD11c+ macrophages were incubated with iC3b, after which transforming growth factor-β (TGF-β) expression was quantified with enzyme-linked immunosorbent assay. In a separate experiment, CD11c+ macrophages were cultured using a Transwell co-culture system with hGECs. After incubation with iC3b, western blot was performed for periostin, fibronectin, Col1a1, TGF-β and β-actin in hGECs. Western blot was also performed for TGF-β in co-cultured CD11c+ macrophages. Periostin and fibronectin expression in co-cultured hGECs were visualized with immunofluorescence. Two-sample t-tests were used for comparisons. A full description of the *in vitro* experiments is available in the Supplementary Methods.

## RESULTS

### Clinical characteristics

**Figure 1:**
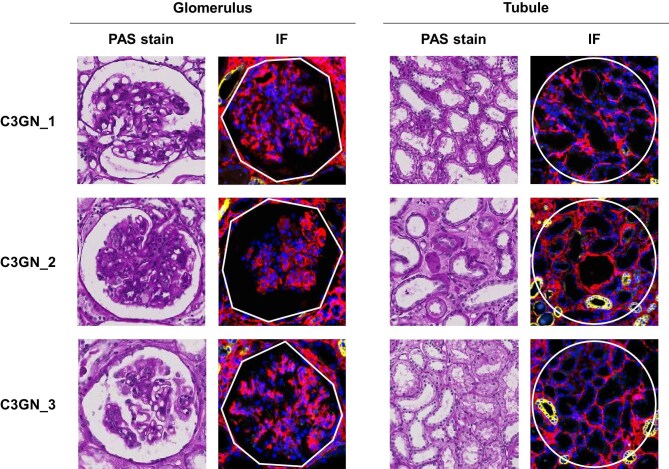
Representative images of the glomeruli and tubules used for spatial transcriptomic analysis. Periodic acid–Schiff stain (left) and correspondingly selected regions of interest in GeoMx (right). Nuclear (blue), PanCK (yellow) and α-SMA (red) were stained to visualize the morphology of each glomerulus.

Three cases of C3G were included in the study, as were 7 donor controls and 41 disease controls consisting of MCD, MN, FSGS and DN. The clinical characteristics and pathological features of the C3G cases are described in Table 1. All patients were male with a mean age of 61 years, presenting with proteinuria and variable degrees of azotemia, edema and hematuria. While the proportion of sclerotic glomeruli varied, there were sufficient non-sclerotic glomeruli for analysis. Representative images are shown in Fig. 1. Over a 5-year follow-up, renal function was stable in one patient, while eGFR declined by 47% in another patient. The third patient was referred out after 1 year of stable renal function. Clinical characteristics of the controls are summarized in Table 2. All healthy donor controls had preserved eGFR and no proteinuria. Among disease controls, 33 of 41 (80%) had eGFR ≥45 mL/min/1.73 m^2^, and 32 of 41 (78%) had a spot urine protein–creatinine ratio ≥3.0.

**Table 1:** Clinical characteristics and histopathological features of C3G cases included in the study.

	Patient 1	Patient 2	Patient 3
Clinical characteristics			
Sex	Male	Male	Male
Age, years	60s	60s	50s
Presentation	Generalized edema	Aggravated azotemia	Generalized edema
	Hematuria	Proteinuria	Hematuria
BUN (mg/dL)	25	25	27
Cr (mg/dL)	1.21	1.84	0.92
eGFR (mL/min/1.73 m^2^)	63	38	91
Hemoglobin (g/dL)	14.8	9.9	9.3
Albumin (g/dL)	3.2	3.2	2.0
Glucose (mg/dL)	106	180	97
Proteinuria	Yes	Yes	Yes
Spot urine protein–creatinine ratio (g/g)	2.8	5.4	2.7
Hematuria	Yes	No	Yes
Hypertension	Yes	Yes	No
Diabetes mellitus	No	Yes	No
Other comorbidities	Gout	Liver cirrhosis, hepatocellular carcinoma	Liver cirrhosis, hepatocellular carcinoma
eGFR at follow-up (mL/min/1.73 m^2^)	65 after 5 years	18 after 5 years	94 after 1 year
Histopathological features			
Cellularity of glomerulus	Diffuse mild hypercellularity (endothelial, mesangial)	Diffuse marked hypercellularity (mesangial)	Diffuse moderate hypercellularity (endothelial, mesangial)
Number of glomeruli	26	19	29
Global sclerosis, *N* (%)	1 (3.8)	14 (73)	4 (13)
Segmental sclerosis, *N* (%)	(–)	(–)	11 (37)
Immunohistochemical stain for C3	3+ in mesangium and periphery	2+ in periphery	2+ in periphery
Electron-dense deposits	Small mesangial, some subendothelial, a few subepithelial	Small mesangial, small subendothelial	Small subendothelial, many subepithelial

BUN: blood urea nitrogen; Cr: creatinine; *N*: number.

**Table 2:** Clinical characteristics of donor and disease controls included in the study.

	Healthy donor	MCD	MN	FSGS	DN
Number of cases	7	13	16	6	6
Age (years)	57 ± 6	48 ± 12	52 ± 10	57 ± 5	56 ± 10
Female, *n* (%)	1 (14.2)	4 (30.8)	3 (18.8)	2 (33.3)	0 (0)
eGFR (mL/min/1.73 m^2^), *n* (%)	89 (88–95)	75 (48–101)	99 (85–107)	85 (80–93)	51 (40–62)
≥90	3 (42.8)	6 (46.2)	11 (68.8)	2 (33.3)	1 (16.7)
≥60 and <90	3 (42.8)	2 (15.4)	3 (18.8)	3 (50.0)	1 (16.7)
≥45 and <60	1 (14.2)	2 (15.4)	1 (6.3)	0 (0)	1 (16.7)
≥30 and <45	0 (0)	3 (23.1)	1 (6.3)	1 (16.7)	3 (50.0)
Hemoglobin (g/dL)	15.0 (14.0–15.3)	12.8 (12.3–14.3)	13.0 (11.7–13.5)	13.4 (11.0–14.7)	11.3 (10.1–12.5)
Albumin (g/dL), *n* (%)	4.0 (3.8–4.3)	2 (2.0–2.4)	2.5 (1.8–2.8)	2.4 (2.0–3.6)	4 (3.5–4.4)
≥3.0	7 (100)	1 (7.7)	3 (18.8)	4 (66.6)	5 (83.3)
<3.0	0 (0)	12 (92.3)	13 (81.2)	2 (33.3)	1 (16.7)
Glucose (mg/dL)	129 (118–133)	98 (92–121)	98 (91–110)	99 (89–126)	127 (119–149)
Spot urine protein–creatinine ratio (g/g), *n* (%)	N/A	9.3 (6.9–11.3)	7.4 (4.5–9.0)	4.5 (1.7–6.0)	2.7 (1.6–3.1)
≥3.0		12 (92.3)	14 (87.5)	4 (66.6)	2 (33.3)
<3.0		1 (7.7)	2 (12.5)	2 (33.3)	4 (66.6)

Age is shown as mean ± standard deviation; laboratory values are presented as median (interquartile range). *n*, number.

### Spatial transcriptomics profiling

The profiling included 151 glomerular and 51 tubulointerstitial ROIs (3 glomerular and 1 tubulointerstitial ROIs per specimen, except for one MN case with 1 glomerular ROI because of a lack of appropriate glomeruli). In a median (interquartile range) surface area of 31 419 (8648–67 633) and 126 746 (52 430–131 871) µm^2^ for each glomerular and tubulointerstitial ROI, a median of 134 (20–250) and 457 (237–709) cells per ROI were identified, and 104 475 (59 246–232 630) reads per ROI were aligned for RNA-seq. RNA-seq saturation was 87.9 (81.9–93.4) %/ROIs. After removal of 58 genes expressed in <50% of total samples, 18 677 remained. Glomerulus marker genes were highly enriched in the targeted glomerular regions (Supplementary data, Fig. S1).

### DEGs between C3G and donor control

**Figure 2:**
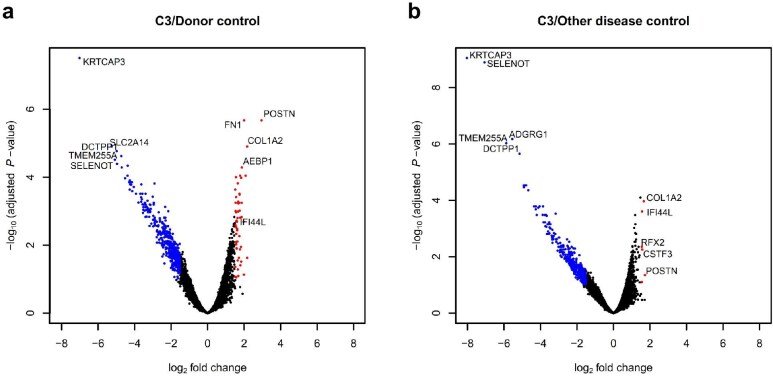
Volcano plots of DEGs for glomerular transcriptional profile of C3G compared with (**a**) healthy donor controls and (**b**) other glomerular disease controls. Fold changes in glomerular gene expressions are shown with upregulated and downregulated DEGs with absolute log2 fold change above 1.5 in red and blue, respectively.

**Figure 3:**
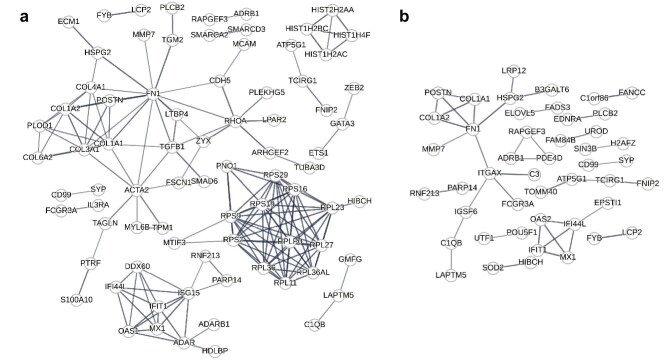
Protein–protein interaction analysis of upregulated DEGs in C3G relative to (**a**) healthy donor controls and (**b**) other glomerular disease controls. The mapping was performed using the STRING database, and DEGs with at least one significant interaction were displayed in the graph. The thickness of each edge reflects the confidence score for the corresponding interaction.

DEG analysis was performed between the three C3G samples and the seven healthy donor controls. Compared with the glomeruli of donor controls, 229 genes were upregulated and 563 were downregulated in the glomeruli of C3G (Fig. 2a, Supplementary data, Table S1), while comparison of tubules showed no significant DEGs. Among the upregulated glomerular DEGs, the greatest fold differences were identified in periostin (*POSTN*) and collagen type I alpha 2 chain (*COL1A2*). Gene Ontology annotations for the upregulated DEGs revealed significant enrichment of multiple molecular function terms including structural molecule activity (GO:0005198), extracellular matrix (ECM) structural constituent (GO:000520), structural constituent of ribosome (GO:0003735), platelet-derived growth factor binding (GO:0048407), growth factor binding (GO:0019838) and protease binding (GO:0002020) (Table 3, Supplementary data, Table S2). Network analysis of the DEGs revealed four main clusters: ECM-related, ribosomal, histone-related and interferon (IFN) activity-related (Supplementary data, Fig. S2a). The majority of the DEGs in these prominent clusters were upregulated, as can be seen in the network diagram restricted to upregulated DEGs (Fig. 3a). Genes *KRTCAP3* (keratinocyte associated protein 3), *SLC2A14* (solute carrier family 2 member 14) and *TMEM255A* (transmembrane protein 255) were among the significantly downregulated with high fold changes, but Gene Ontology analysis did not reveal significant functional enrichment.

**Table 3:** Top Gene Ontologies among glomerular DEGs upregulated in C3G—for each comparison, up to 10 Gene Ontology terms with the lowest false-discovery rate for each domain are presented with a sample of up to 10 individual annotated genes reported by the ToppGene Suite.

C3G against	Domain	Gene Ontology terms (up to 10 per domain)	False-discovery rate	Sample of annotated genes (up to 10)
Donor controls	Molecular function	GO:0005198 (structural molecule activity)	1.56E-07	*TPM1, RPL27, RPL36AL, RPLP0, RPS2, RPS9, RPS16, RPS19, RPS29, RPL23*
		GO:0005201 (ECM structural constituent)	8.56E-05	*AEBP1, LTBP4, COL1A1, COL1A2, COL3A1, COL4A1, HSPG2, COL6A2, FBLN5, FN1*
		GO:0003735 (structural constituent of ribosome)	5.37E-04	*RPL27, RPL36AL, RPLP0, RPS2, RPS9, RPS16, RPS19, RPS29, RPL23, RPL36*
		GO:0048407 (platelet-derived growth factor binding)	1.22E-03	*COL1A1, COL1A2, COL3A1, COL4A1*
		GO:0019838 (growth factor binding)	1.97E-03	*RPS2, AXL, RPS19, CHRDL1, LTBP4, COL1A1, COL1A2, COL3A1, COL4A1, IL3RA*
		GO:0030020 (ECM structural constituent conferring tensile strength)	1.19E-02	*COL1A1, COL1A2, COL3A1, COL4A1, COL6A2*
		GO:0002020 (protease binding)	2.17E-02	*MARCHF6, SERPINA1, COL1A1, COL1A2, COL3A1, HSPG2, FLOT2, FN1, ECM1*
		GO:0019843 (rRNA binding)	2.62E-02	*RPLP0, RPS9, RPL23, MRPL18, CAVIN1, RPL11*
	Biological process	GO:0001568 (blood vessel development)	8.89E-05	*CYBB, TNFSF12, ACTA2, ETS1, MCAM, RPS29, PLEKHG5, ADRB1, RAPGEF3, PDCL3*
		GO:0001944 (vasculature development)	8.89E-05	*CYBB, TNFSF12, ACTA2, ETS1, MCAM, RPS29, PLEKHG5, ADRB1, RAPGEF3, PDCL3*
		GO:0048514 (blood vessel morphogenesis)	7.13E-04	*CYBB, TNFSF12, ETS1, MCAM, RPS29, PLEKHG5, ADRB1, RAPGEF3, PDCL3, NOTCH3*
		GO:0072359 (circulatory system development)	8.80E-04	*CYBB, TPM1, TNFSF12, ACTA2, ETS1, GATA3, MCAM, MECOM, RPS29, PLEKHG5*
		GO:0001525 (angiogenesis)	1.63E-03	*CYBB, TNFSF12, ETS1, MCAM, RPS29, PLEKHG5, RAPGEF3, PDCL3, NOTCH3, COL4A1*
		GO:0035239 (tube morphogenesis)	1.80E-03	*CYBB, TNFSF12, ETS1, GATA3, MCAM, MECOM, RPS29, PLEKHG5, ZEB2, MEIS2*
		GO:0048646 (anatomical structure formation involved in morphogenesis)	3.01E-03	*CYBB, TPM1, TNFSF12, ETS1, GATA3, MCAM, TCIRG1, RPS29, PLEKHG5, ZEB2*
		GO:0034097 (response to cytokine)	3.12E-03	*RPLP0, SHFL, RPS2, AXL, GATA3, TCIRG1, RPS16, ADAR, ZYX, LAPTM5*
		GO:0009607 (response to biotic stimulus)	3.12E-03	*CYBB, CFHR1, SHFL, AXL, ACTA2, GATA3, MECOM, RPS19, ADAR, ADARB1*
		GO:0051707 (response to other organism)	3.32E-03	*CYBB, CFHR1, SHFL, AXL, ACTA2, GATA3, MECOM, RPS19, ADAR, ADARB1*
	Cellular component	GO:0031012 (ECM)	1.23E-05	*ATP5MC1, AXL, ETS1, GATA3, MECOM, ZEB2, ADRB1, ATXN1, FDPS, CXCL9*
		GO:0030312 (external encapsulating structure)	1.23E-05	*CYBB, AXL, ETS1, GATA3, TCIRG1, MECOM, RPS19, ZEB2, ZYX, SERPINA1*
		GO:0062023 (collagen-containing ECM)	1.23E-05	*ETS1, GATA3, RPS19, ADAR, GPX1, SIGLEC16, OAS1, WFDC1, PARP14, TGFB1*
		GO:0022626 (cytosolic ribosome)	1.23E-05	*AXL, ADAR, LAPTM5, CASP4, OAS1, PARP14, ECM1, PIAS4, ISG15*
		GO:0005925 (focal adhesion)	2.86E-05	*TNFSF12, ACTA2, ETS1, GATA3, PLEKHG5, ZEB2, TAGLN, HSPG2, FN1, GPX1*
		GO:0030055 (cell–substrate junction)	3.60E-05	*RHOA, CDH5*
		GO:0005581 (collagen trimer)	7.59E-05	*RPS9, SLC40A1*
		GO:0044391 (ribosomal subunit)	1.16E-04	*GATA3, ADARB1, ZEB2, S100A4, COL1A1, HSPG2, TBX3, FN1, WNT11, TGFB1*
		GO:0005840 (ribosome)	6.04E-04	*CFHR1, AXL, TCIM, ETS1, GATA3, RPS19, ADAR, MEIS2, LAPTM5, PIGR*
		GO:0070161 (anchoring junction)	1.39E-03	*AXL, LAPTM5, SPON2, CASP4, TGFB1*
Disease controls	Molecular function	GO:0002020 (protease binding)	0.0451	*MAGEA4, HSPG2, FN1, SERPINB9, NFRKB, TNFAIP3, COL1A1, COL1A2*
	Biological process	None		
	Cellular component	GO:0005584 (collagen type I trimer)	0.0228	*COL1A1, COL1A2*
All controls	Molecular function	None		
	Biological process	None		
	Cellular component	GO:0005584 (collagen type I trimer)	0.0241	*COL1A1, COL1A2*

### DEGs between C3G and other major glomerulonephritis cases

Analysis of C3G compared with other major glomerulonephritis cases showed 157 upregulated and 347 downregulated DEGs in the glomeruli (Fig. 2b, Supplementary data, Table S1), and no significant DEGs were found between the tubules. While downregulated glomerular DEGs showed no significant functional gene enrichment, upregulated glomerular DEGs showed enrichment of the Gene Ontology term protease binding (GO:0002020) (Table 3, Supplementary data, Table S2). The greatest fold differences among the upregulated DEGs were found in *POSTN, COL1A2*, cleavage stimulation factor subunit 3 (CSTF3), IFN induced protein 44 like (*IFI44L*) and regulatory factor X2 (*RFX2*), of which all but CSTF3 were also upregulated in comparison with the donor controls. Network analysis of the DEGs showed clusters of ECM proteins and proteins related to IFN activity (Supplementary data, Fig. S2b), which were preserved in the compact network diagram restricted to upregulated DEGs (Fig. 3b). Notably, C3 showed a high probability of interaction with ITGAX (integrin subunit alpha X), which in turn had linkage to the cluster of ECM proteins through a high-probability interaction with FN1 and was connected to C1QB (complement C1q B chain) through IGSF6 (immunoglobulin superfamily member 6).

When C3G cases were compared with donor and disease controls combined, 163 upregulated and 367 downregulated DEGs were identified in the glomeruli of C3G, and the top five upregulated and downregulated DEGs in terms of fold difference were identical to those from disease controls alone (Supplementary data, Fig. S3 and Table S1). The only significant functional enrichment was for collagen type I trimer (GO:0005584) among the upregulated glomerular DEGs (Table 3, Supplementary data, Table S2). Network analysis of DEGs also prominently showed ECM components and IFN-related proteins (Supplementary data, Fig. S4).

### Protein level validation of DEGs

**Figure 4:**
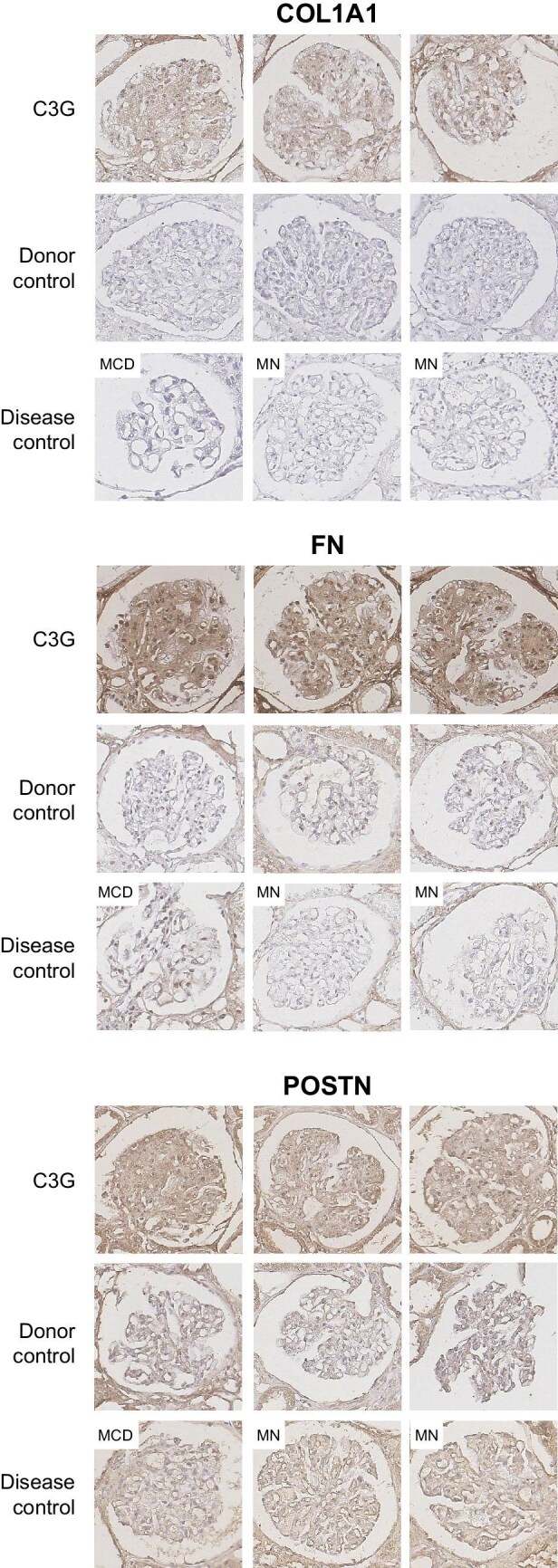
Immunohistochemical stain for proteins corresponding to upregulated glomerular DEGs in C3G. Stains for proteins Col1a1, fibronectin and periostin were compared among patient biopsy slides for C3G, healthy donor controls and glomerular disease controls that include MCD and MN. Magnification: 400×.

We validated key DEGs with high fold differences at the protein level using immunohistochemistry for Col1a1, fibronectin and periostin in C3G and controls (Fig. 4, Supplementary data, Fig. S5). Col1a1 and fibronectin were negligible in controls but significantly stained in C3G glomeruli, especially in the mesangium. Periostin showed strong staining in C3G, likely in mesangial cells and podocytes, with weaker expression in disease controls and minimal in healthy donors.

### *In vitro* assessment of CD11c in modulation of glomerular fibrosis

**Figure 5:**
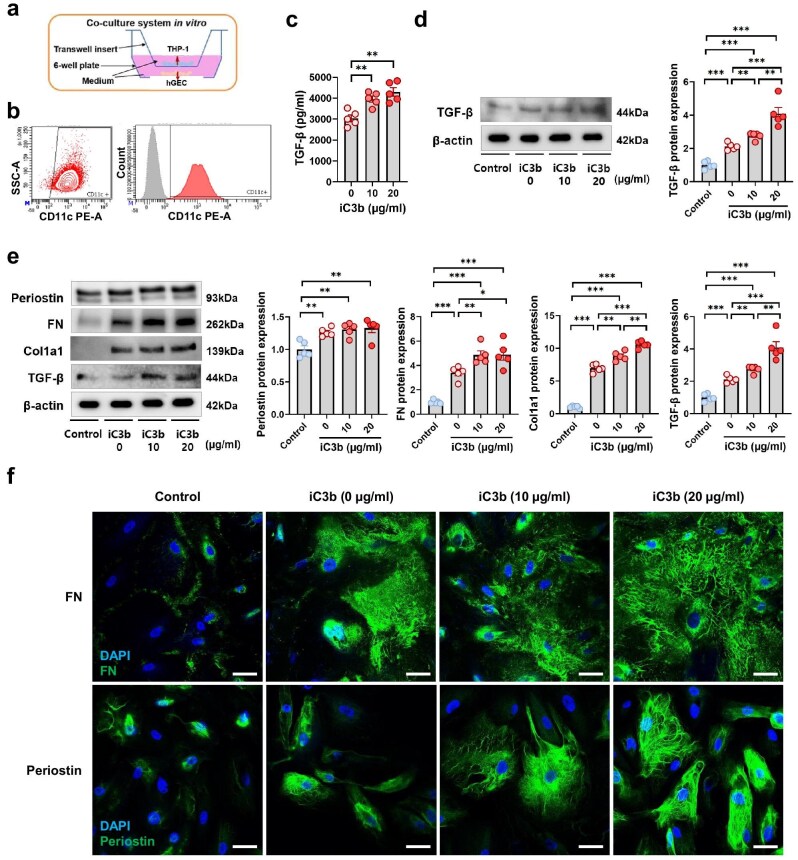
Expression of ECM components and TGF-β in hGECs co-cultured with iC3b-stimulated CD11c+ macrophages. (**a**) Diagram of the *in vitro* Transwell co-culture system. (**b**) Fluorescence-activated cell sorting for isolation of CD11c+ macrophages. (**c**) TGF-β expression in CD11c+ macrophages after 48-h incubation with iC3b (0, 10, 20 µg/mL), quantified with enzyme-linked immunosorbent assay. Two-sample t-tests between groups were performed, where: ^*^P < .05, ^**^P < .01 and ^***^P < .001. (**d**) TGF-β expression in CD11c+ macrophages after 48-h incubation with iC3b (0, 10, 20 µg/mL) in co-culture with hGECs, including a negative control without co-cultured hGECs, quantified from western blot images. (**e**) ECM component and TGF-β expression in hGECs after 48-h incubation with iC3b (0, 10, 20 µg/mL) in co-culture with CD11c+ macrophages, including a negative control without the macrophages. (**f**) Immunofluorescence staining for FN and periostin expression in hGECs co-cultured with CD11c+ macrophages. Scale bars = 50 μm.

Based on the potential interaction between C3 and ECM component proteins through ITGAX, also known as CD11c, in the network analysis of glomerular DEGs upregulated in C3G, we investigated whether stimulation of CD11c with C3 can modulate glomerular fibrosis using an *in vitro* co-culture model (Fig. 5a). CD11c+ macrophages were isolated from THP-1 human monocyte cell line via flow cytometry (Fig. 5b). After stimulation with iC3b, the major ligand of CD11c/CD18, CD11c+ macrophages showed significantly higher expression of the TGF-β protein (Fig. 5c). CD11c+ macrophages also exhibited dose-dependent increases in TGF-β expression after iC3b stimulation in a co-culture with hGECs (Fig. 5d). Co-cultured hGECs showed greater expression of ECM component proteins periostin, fibronectin and collagen type I alpha 1 (Col1a1) as well as TGF-β relative to negative controls (Fig. 5e). Fibronectin, Col1a1 and TGF-β expression levels were significantly higher under iC3b stimulation, with up to 10-fold increase of Col1a1 expression in a dose-dependent manner, while periostin was constitutively expressed at similar levels. Co-cultured hGECs also showed strong immunofluorescence staining for fibronectin and periostin unlike control hGECs, and iC3b stimulation appeared to enhance their expression (Fig. 5f).

## DISCUSSION

In this study, we explored the substructure-specific transcriptional profile of C3G relative to healthy controls and other glomerular diseases to gain potential insights into the pathophysiology of C3G. Gene Ontology enrichment analysis of the glomerular DEGs suggested that functional enrichment of genes with protease binding function may be characteristic of C3G. Moreover, clusters of ECM proteins and IFN-related proteins were consistently identified in the protein–protein interaction network among the upregulated glomerular DEGs in C3G. These ECM components were also relatively highly expressed at the protein level in C3G. Compared with other glomerular disease controls, C3G showed glomerular upregulation of complement C3 with possible interactions with ITGAX (CD11c), a likewise upregulated integrin subunit which in turn had potential interactions with other highly enriched ECM-related proteins.

The observed pattern of DEGs, dominated by upregulation of ECM proteins, appears to be consistent with the typical histopathological features of C3G. Upregulated glomerular DEGs with the highest fold changes common to both comparisons were ECM-related proteins POSTN and COL1A2, consistent with the membranoproliferative pattern of injury associated with C3G [17, 18]. While ECM components may reflect glomerular fibrosis in advanced kidney disease, exclusion of samples with low eGFR, selection of non-sclerotic glomeruli, and consistent results found in comparison with other disease controls altogether make it less likely that their upregulation is a mere consequence of the chronic, nonspecific sclerotic process. The immunohistochemistry results also show marked upregulation of ECM components in non-sclerotic glomeruli of C3G.

Beyond individual DEGs, comparative analysis of the functional enrichments in C3G relative to the two control groups suggests additional processes potentially involved in C3G. The Gene Ontology term protease binding (GO:0002020) was significantly enriched in both comparisons, and among the implicated genes include ECM-related proteins such as FN1 and COL1A1/2 as well as intracellular proteins such as NFRKB and SERPINB9 that regulate or inhibit proteases. While these proteins may contribute to distinct pathways, ECM proteins do interact with matrix metalloproteinases (MMPs), which not only degrade the ECM but also regulate homeostasis and inflammation in the kidney [19]. MMP7, among the consistently upregulated glomerular DEGs and linked to FN1 in the network analysis, may therefore be involved in the pathophysiology of C3G. Additionally, multiple functional enrichments are only observed among upregulated DEGs relative to donor controls, including Gene Ontology terms blood vessel development (GO:0001568) and vascular development (GO:0001944) with very low false-discovery rates. While not necessarily C3G-specific, they may still indicate universal processes in C3G pathophysiology. In this case, enrichments related to the vasculature are consistent with the known central role of the endothelium in C3G.

The possible interaction between C3 and ECM proteins suggested by the network analysis could be relevant to the pathophysiology of C3G. In C3G, C3 is highly expressed in the glomeruli [5], but the role of the ECM has not been fully characterized in the existing literature. Dysregulation of the alternative pathway in C3G most frequently occurs in the “fluid phase” of plasma proteins, which alter the glomerular microenvironment including endothelial cells and the ECM [1, 5]. ITGAX associates with CD18 to form CD11c/CD18, a beta-2 integrin-type complement receptor known as complement receptor 4 (CR4) [20]. Found on leukocytes, CR4 binds to iC3b and is involved in adhesion, migration and phagocytosis [21]. Complement receptor 3 (CR3), in the same family and often co-expressed with CR4 [22], has been studied as a potential downregulator of inflammation in C3G through interactions with iC3b [23]. While CR4 has been less studied, we observed *in vitro* that exposure of CD11c+ macrophages to iC3b, potentially stimulating CR4 activity, not only leads to greater expression of fibrotic response marker TGF-β from these macrophages but also shows increased expression of TGF-β and ECM-related proteins in co-cultured hGECs. Our result suggests the possibility that CR4, both a complement receptor and an integrin, is involved in the modulation of glomerular inflammation and fibrosis in C3G.

Periostin, typically absent in normal kidney tissues, is an ECM component linked to kidney inflammation and fibrosis [24, 25]. Its glomerular upregulation in C3G suggests ongoing fibrosis and inflammation. *In vitro* validation with CD11c+ macrophages showed iC3b-independent periostin overexpression, indicating a role beyond fibrosis. Recently, periostin was also identified as a mesangial cell marker [26], suggesting mesangial expansion and ECM remodeling may contribute to its upregulation in C3G.

Also among the consistently upregulated DEGs in both comparisons were IFN-related genes with no clear known function in C3G. Namely, the genes *IFI44L, IFIT1, MX1* and *OAS2* all encode proteins involved in IFN-mediated antiviral responses [27]. Activation of type 1 IFN (IFN-I) pathway has been implicated in autoimmune diseases including lupus nephritis, where IFN-I produced by renal resident cells promote glomerular fibrosis [28]. A recent analysis identified IFI44, a paralog of IFI44L, as one of the potential key biomarkers of LN [29]. Although IFN-I has not been previously associated with C3G, our results suggest that IFN-related signals could also have pro-inflammatory roles in C3G.

This study has several limitations. First, the limited reads per ROI likely reduced sensitivity and prevented cell-type analysis. More samples could reveal additional DEGs, particularly those with low baseline expression. Second, key DEGs like CD11c were not validated at the protein level due to quantification challenges in the glomerulus. Additionally, our *in vitro* co-culture model with THP-1 macrophages and glomerular endothelial cells may not fully replicate *in vivo* macrophage–glomerular interactions, especially given cell line differences and complement dysregulation in C3G. However, spatial transcriptomics may have captured early mRNA-level pathological changes. Third, as our study was based on three male Korean C3G cases, selection bias may exist. Finally, single-cell level analysis was limited due to the rarity of C3G and the scarcity of fresh kidney biopsy samples. Moreover, single-cell RNA sequencing often captures few glomerular cells, making it less ideal for studying intraglomerular transcriptomic changes in a rare disease like C3G.

In conclusion, we explored the spatial transcriptomic profile of C3G and identified, among DEGs in the glomerulus, consistent enrichment of genes related to the ECM and to IFN activity. As the first report of kidney substructure-specific transcriptomic profile of C3G to date, our study suggests these genes may have a previously underrecognized role in the complement-mediated pathogenesis of C3G.

## Supplementary Material

sfaf139_Supplemental_Files

## Data Availability

The complete transcriptomics data reported in this study are openly available on Figshare at doi:10.6084/m9.figshare.25249261.
