# Correction to: Spatially resolved transcriptomic profiling for glomerular and tubulointerstitial gene expression in C3 glomerulopathy

**DOI:** 10.1093/ckj/sfaf257

**Published:** 2025-09-16

This is a correction to: Jung Hun Koh, Minji Kang, Sehoon Park, Ha Yeon Shin, Hyunah Ku, Seong Min Lee, Jeong Min Cho, Semin Cho, Yaerim Kim, Soojin Lee, Hajeong Lee, Kwon-Wook Joo, Kyung Chul Moon, Seung Hee Yang, Hyun Je Kim, Dong Ki Kim, on behalf of the KORNERSTONE investigators, Spatially resolved transcriptomic profiling for glomerular and tubulointerstitial gene expression in C3 glomerulopathy, *Clinical Kidney Journal*, Volume 18, Issue 5, May 2025, sfaf139, https://doi.org/10.1093/ckj/sfaf139

The originally published version of this manuscript omitted to designate Seung Hee Yang as a co-corresponding author. This error has been corrected, and two additional funding sources have been added to the funding statement.

