# Supplementary material for: Spatially resolved transcriptomic profiling for glomerular and tubulointerstitial gene expression in C3 glomerulopathy
Source: Clin Kidney J. 2025 May 8;18(5):sfaf139. doi: 10.1093/ckj/sfaf139 (PMC12102689; doi:10.1093/ckj/sfaf139)
Supplement: sfaf139_Supplemental_Files [file sfaf139_supplemental_files.zip › Supplementary_Material_R2.pdf]

SUPPLEMENTARY MATERIAL

TABLE OF CONTENTS

Supplementary Methods

Slide preparation and processing

In vitro validation experiments

Supplementary Figure 1. Expression of glomerulus marker genes (EHD3, NPHS1, WT1) and tubule marker genes (AQP2, FXYD2, UMOD) in glomerular and tubulointerstitial regions of interest (ROIs).

Supplementary Figure 2. Protein-protein interaction analysis of differentially expressed genes (DEGs) in C3 glomerulopathy.

Supplementary Figure 3. Volcano plots of differentially expressed genes (DEGs) for glomerular transcriptional profile of C3 glomerulopathy compared to all controls.

Supplementary Figure 4. Protein-protein interaction analysis of differentially expressed genes (DEGs) in C3 glomerulopathy relative to all controls.

Supplementary Figure 5. Negative controls for immunohistochemical stains.

Supplementary Table 1. All differentially expressed genes (DEGs) between C3 glomerulopathy (C3G) cases and healthy donor controls and between C3G cases and other glomerulonephritis controls by DESeq2 method. ***Provided as a separate MS Excel file.***

Supplementary Table 2. Significant gene ontologies among upregulated differentially expressed genes (DEGs) as annotated by the ToppGene Suite.

Supplementary Methods

Slide preparation and processing

Kidney tissues were mounted on Leica BOND Plus slides (Leica Biosystems) with thicknesses of 5 μm. The slides were baked at 60℃ for 30 minutes, deparaffinized with CitriSolv (Decon Laboratories), and gradually rehydrated with ethanol and 1X phosphate-buffered saline (PBS). To unmask the antigen epitopes, the slides were dipped in 1X Tris-EDTA (pH 9.0) and incubated for 15 minutes, followed by RNA target exposure with a 1 μg/mL proteinase K solution at 37℃ for 15 minutes and 1X PBS wash for 5 minutes. For *in situ* hybridization, the slides were incubated overnight at 37℃ with the GeoMx Whole Transcriptome Atlas, which consists of RNA probes conjugated with unique ultraviolet-photocleavable oligonucleotide barcodes targeting 18,677 genes. The slides were subsequently washed then stained with the following morphology markers for 1 hour at room temperature: SYTO 13 (Nanostring, 121300303), Pan-cytokeratin (Novus, NBP2-33200 AF594), and alpha-smooth muscle actin (Abcam, ab202296).

The slides were loaded into the GeoMx Digital Spatial Profiler (DSP) instrument, and representative glomerular and tubulointerstitial substructures were selected as regions of interest (ROIs) by a kidney pathologist. The oligonucleotide barcodes for the target genes within each ROI were photocleaved and collected into a DSP collection plate. The oligonucleotides were amplified through polymerase chain reaction (PCR) with primer pairs and i5 and i7 dual-indexing sequences. The PCR products were pooled and purified with AMPure XP Reagent (A63880) to obtain the sequencing library. Following quality assessment using the Agilent 4150 TapeStation system, they were sequenced on an Illumina NovaSeq 6000 with 27 × 27 paired-end reads.

In vitro validation experiments

The expressions of periostin, collagen type I alpha 1, and fibronectin in C3G, donor controls, and glomerular disease controls (MCD and MN) included in the transcriptomic profiling were assessed with immunohistochemistry as follows. Paraffin-embedded sections (5 μm thick) were deparaffinized in xylene and rehydrated through a graded ethanol series. Antigen retrieval was performed by microwaving the sections in 0.1 M sodium citrate buffer (pH 6.0). Endogenous peroxidase activity was inhibited with 0.3% hydrogen peroxide diluted in methanol for 15 minutes. Sections were then blocked with a solution containing 0.03% Triton X-100, 5% BSA, and 10% goat serum at 25 °C for 30 minutes, followed by incubation with primary antibodies against fibronectin (1:100, sc-18825, Santa Cruz), periostin (1:300, ab14014, Abcam), and Col1a1 (1:100, sc-293182, Santa Cruz) at 4 °C for 24 hours. Afterward, horseradish peroxidase-labeled polymer secondary antibodies anti-rabbit (K4003, Agilent DAKO) and anti-mouse (K4001, Agilent DAKO) were applied and incubated for 1 hour at 25 °C. Mayer's hematoxylin (Sigma-Aldrich) was applied for nuclear staining. The staining procedures were also performed without primary antibodies to serve as negative controls. The stained areas were visualized and quantified using a Leica inverted microscope (Leica Camera) and analyzed with the LAS-4000 software (Leica Camera).

Next, macrophages were isolated from THP-1 monocyte cell line with MojoSort Isolation Kits (Biolegend), and CD11c+ macrophages were selected with flow cytometry. Human glomerular endothelial cells (hGECs) were collected from unaffected tissues of nephrectomy specimens from renal cell carcinoma patients, as described previously. CD11c+ macrophages were cultured at a density of 106 cells per well and incubated with purified human iC3b (Sigma-Aldrich, Cat. 204863) at concentrations of 0, 10, and 20 μg/ml. After 48 hours, the macrophages were harvested for quantification of transforming growth factor-β (TGF-β)

expression with ELISA. In a separate experiment, CD11c+ macrophages were cultured using a Transwell co-culture system with hGECs plated in the lower compartment. CD11c+ macrophages and hGECs were also cultured in isolation as negative controls. The Transwell assays were incubated with iC3b at concentrations of 0, 10, and 20 μg/ml for 48 hours. The hGECs were then harvested, protein was extracted, and Western blot was performed for periostin, fibronectin, collagen type I alpha 1, TGF-β, and β-actin. Western blot was also performed for TGF-β in co-cultured CD11c+ macrophages. Two-sample t-tests were used for comparisons. The following primary antibodies were used: periostin (Abcam, Ab152099), fibronectin (Abcam, Ab2413), collagen type I alpha 1 (GeneTex, GTX112731), TGF-β (Proteintech, 21898-1-AP), and β-actin (Sigma-Aldrich, A1978). As for secondary antibodies, anti-rabbit IgG HRP (Cell Signaling Technology, #7074) was used for periostin, fibronectin, collagen type I alpha 1 and TGF-β. Anti-mouse IgG HRP (Cell Signaling Technology, #7076) was used for β-actin.

Co-cultured hGECs were fixed and visualized under confocal microscopy after immunofluorescence staining for periostin and fibronectin. Specifically, a violet laser line (405 nm) was used to detect 4',6-diamidino-2-phenylindole (DAPI) (Thermo Fisher Scientific, emission range: 420-480 nm) for nuclear staining. Primary antibodies against fibronectin (1:100, sc-18825, Santa Cruz) and periostin (1:300, ab14014, Abcam) were incubated for 24 hours at 4 °C. Afterward, sections were incubated for 1 hour at 25 °C with the corresponding secondary antibodies: Alexa Fluor 488-conjugated anti-rabbit (1:600, A-11070, Thermo Fisher Scientific) for periostin and Alexa Flour 488-conjugated anti-mouse (1:600, A-11001, Thermo Fisher Scientific) for fibronectin. These were excited using an argon laser (488 nm) at 5% excitation power and 30% laser output. Emission spectra were acquired using an acousto-optical beam splitter. Confocal scanning was performed at 400 Hz

with a 4x line average and 1 Airy unit (66.57 nm) resolution. Images were captured at a resolution of 1024 x 1024 pixels (XY) in sequential scanning mode using the LAS X software (Leica).

Supplementary Figure 1. Expression of glomerulus marker genes (EHD3, NPHS1, WT1) and tubule marker genes (AQP2, FXYD2, UMOD) in glomerular and tubulointerstitial regions of interest (ROIs). Relative expression is shown as a percentage of total reads from each ROI.

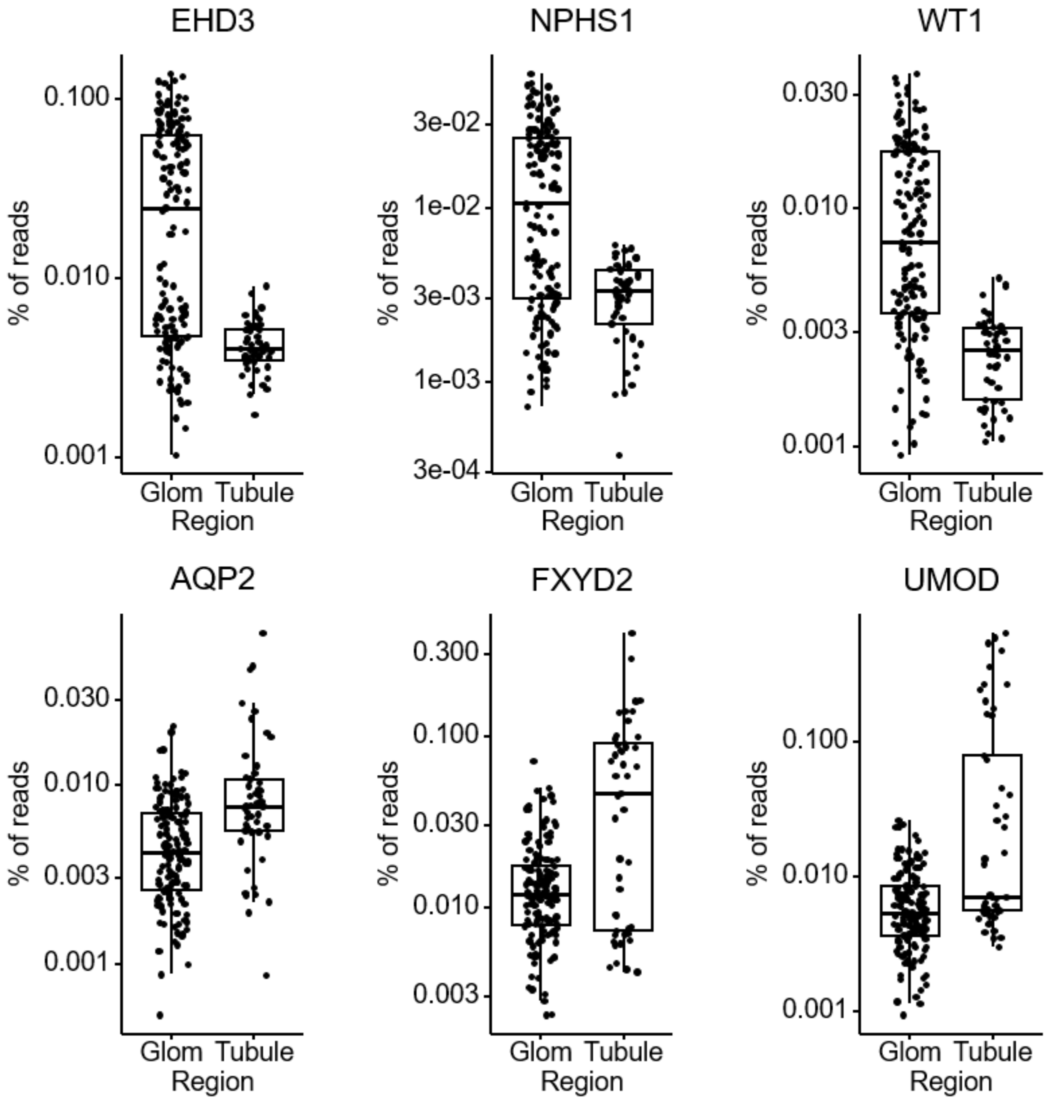

Supplementary Figure 2. Protein-protein interaction analysis of differentially expressed genes (DEGs) in C3 glomerulopathy relative to (a) healthy donor controls and (b) other glomerular disease controls. DEGs with at least one significant interaction are shown. Red and blue nodes correspond to upregulated and downregulated DEGs, respectively.

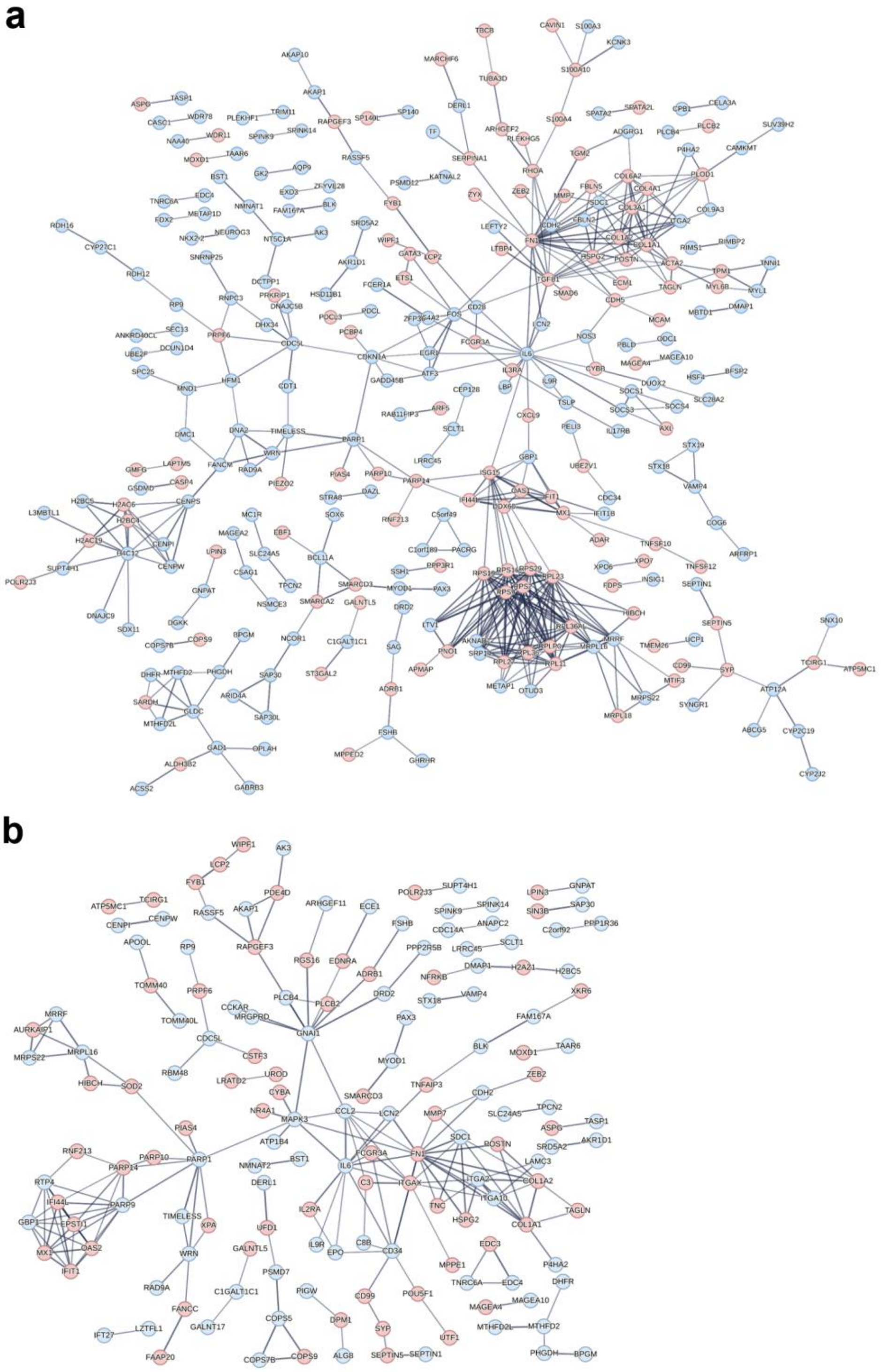

Supplementary Figure 3. Volcano plots of differentially expressed genes (DEGs) for glomerular transcriptional profile of C3 glomerulopathy compared to all controls.

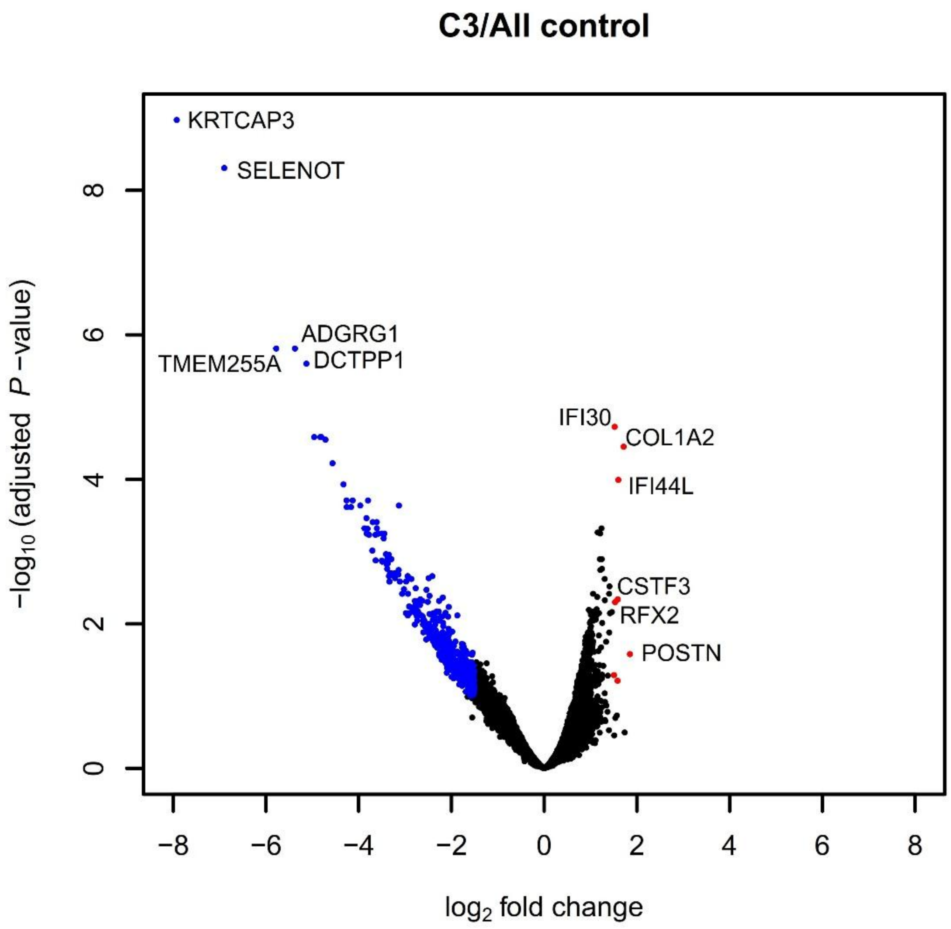

Supplementary Figure 4. Protein-protein interaction analysis of differentially expressed genes (DEGs) in C3 glomerulopathy relative to all controls. DEGs with at least one significant interaction are shown. Red and blue nodes correspond to upregulated and downregulated DEGs, respectively.

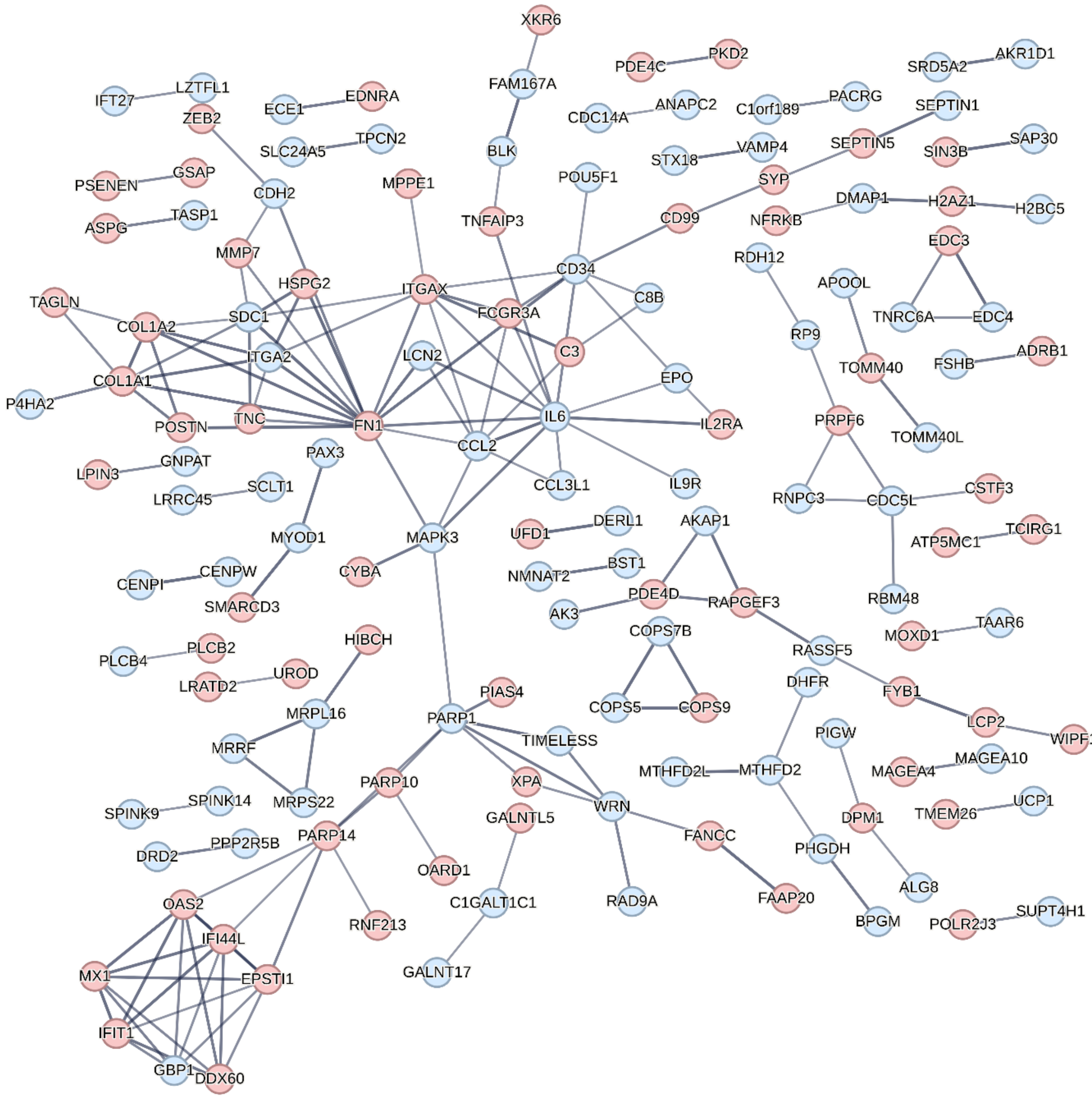

Supplementary Figure 5. Negative controls for immunohistochemical stains. Immunohistochemical staining was performed on kidney biopsy slides of healthy donor controls while omitting the primary antibodies for each target protein.

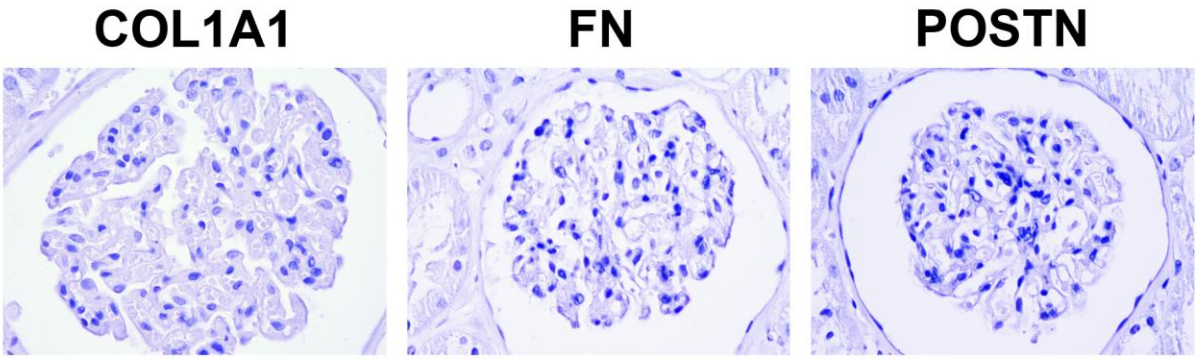

Supplementary Table 1. All differentially expressed genes (DEGs) between C3 glomerulopathy (C3G) cases and healthy donor controls and between C3G cases and other glomerulonephritis controls by DESeq2 method. Log2 fold changes and false discovery rates are presented for each significant DEG.

Provided as a separate MS Excel file.

Supplementary Table 2. Significant gene ontologies among upregulated DEGs as annotated by the ToppGene Suite. For each comparison, all gene ontology terms with the lowest false discovery rate for each domain are presented with all individual annotated genes reported by the ToppGene Suite. (C3G: C3 glomerulopathy)

C3G against	Domain	Gene ontology terms	False discovery rate	Annotated gene lists
donor controls (7)	molecular function	GO:0005198 (structural molecule activity)	1.56E-07	TPM1, RPL27, RPL36AL, RPLP0, RPS2, RPS9, RPS16, RPS19, RPS29, RPL23, H2AC6, H2BC4, AEBP1, H4C12, MYL6B, LTBP4, COL1A1, COL1A2, COL3A1, COL4A1, HSPG2, COL6A2, RPL36, FBLN5, FN1, SEPTIN5, TUBA3D, H2AC19, ECM1, POSTN, MRPL18, MMRN2, CAVIN1, EPB41L2, RPL11
		GO:0005201 (extracellular matrix structural constituent)	8.56E-05	AEBP1, LTBP4, COL1A1, COL1A2, COL3A1, COL4A1, HSPG2, COL6A2, FBLN5, FN1, ECM1, POSTN, MMRN2
		GO:0003735 (structural constituent of ribosome)	5.37E-04	RPL27, RPL36AL, RPLP0, RPS2, RPS9, RPS16, RPS19, RPS29, RPL23, RPL36, MRPL18, RPL11
		GO:0048407 (platelet-derived growth factor binding)	1.22E-03	COL1A1, COL1A2, COL3A1, COL4A1
		GO:0019838 (growth factor binding)	1.97E-03	RPS2, AXL, RPS19, CHRDL1, LTBP4, COL1A1, COL1A2, COL3A1, COL4A1, IL3RA
		GO:0030020 (extracellular matrix structural constituent conferring tensile strength)	1.19E-02	COL1A1, COL1A2, COL3A1, COL4A1, COL6A2
		GO:0002020 (protease binding)	2.17E-02	MARCHF6, SERPINA1, COL1A1, COL1A2, COL3A1, HSPG2, FLOT2, FN1, ECM1
		GO:0019843 (rRNA binding)	2.62E-02	RPLP0, RPS9, RPL23, MRPL18, CAVIN1, RPL11
	biological process	GO:0001568 (blood vessel development)	8.89E-05	CYBB, TNFSF12, ACTA2, ETS1, MCAM, RPS29, PLEKHG5, ADRB1, RAPGEF3, PDCL3, NOTCH3, COL1A1, COL1A2, COL3A1, COL4A1, HSPG2, TBX3, FBLN5, FN1, WNT11, GPX1, GMFG, SOX17, RNF213, NIBAN2, ECM1, TGFB1, RHOA, PPP3R1, MMRN2, LPAR2, TNFAIP2, PEAK1, CDH5, GAA, SMAD6
		GO:0001944 (vasculature development)	8.89E-05	CYBB, TNFSF12, ACTA2, ETS1, MCAM, RPS29, PLEKHG5, ADRB1, RAPGEF3, PDCL3, NOTCH3, COL1A1, COL1A2, COL3A1, COL4A1, HSPG2, TBX3, FBLN5, FN1, WNT11, GPX1, GMFG, SOX17, RNF213, NIBAN2, ECM1, TGFB1, RHOA, PPP3R1, SMARCA2, MMRN2, LPAR2, TNFAIP2, PEAK1, CDH5, GAA, SMAD6
		GO:0048514 (blood vessel morphogenesis)	7.13E-04	CYBB, TNFSF12, ETS1, MCAM, RPS29, PLEKHG5, ADRB1, RAPGEF3, PDCL3, NOTCH3, COL3A1, COL4A1, HSPG2, FBLN5, FN1, WNT11, GPX1, GMFG, SOX17, RNF213, NIBAN2, ECM1, TGFB1, RHOA, PPP3R1, MMRN2, LPAR2, TNFAIP2, PEAK1, CDH5, SMAD6
		GO:0072359 (circulatory system development)	8.80E-04	CYBB, TPM1, TNFSF12, ACTA2, ETS1, GATA3, MCAM, MECOM, RPS29, PLEKHG5, MEIS2, ADRB1, RAPGEF3, FDPS, PDCL3, PLCB2, NOTCH3, COL1A1, COL1A2, COL3A1, COL4A1, HSPG2, TBX3, FBLN5, FN1, WNT11, GPX1, GMFG, SOX17, RNF213, NIBAN2, ECM1, CRIP1, TGFB1, RHOA, PPP3R1, WDR11, SMARCA2, MMRN2, SMARCD3, LPAR2, TNFAIP2, PEAK1, CDH5, GAA, SMAD6
		GO:0001525 (angiogenesis)	1.63E-03	CYBB, TNFSF12, ETS1, MCAM, RPS29, PLEKHG5, RAPGEF3, PDCL3, NOTCH3, COL4A1, HSPG2, FBLN5, FN1, GPX1, GMFG, SOX17, RNF213, NIBAN2, ECM1, TGFB1, RHOA, PPP3R1, MMRN2, LPAR2, TNFAIP2, PEAK1, CDH5
		GO:0035239 (tube morphogenesis)	1.80E-03	CYBB, TNFSF12, ETS1, GATA3, MCAM, MECOM, RPS29, PLEKHG5, ZEB2, MEIS2, ADRB1, RAPGEF3, PDCL3, NOTCH3, COL3A1, COL4A1, HSPG2, TBX3, FBLN5, FN1, WNT11, GPX1, GMFG, SOX17, RNF213, NIBAN2, ECM1, TGFB1, RHOA, TGM2, PPP3R1, MMRN2, LPAR2, TNFAIP2, PEAK1, CDH5, SMAD6
		GO:0048646 (anatomical structure formation involved in morphogenesis)	3.01E-03	CYBB, TPM1, TNFSF12, ETS1, GATA3, MCAM, TCIRG1, RPS29, PLEKHG5, ZEB2, RAPGEF3, PDCL3, CXCL9, NOTCH3, COL1A1, COL3A1, COL4A1, HSPG2, TBX3, FBLN5, FN1, WNT11, GPX1, GMFG, SOX17, RNF213, NPC2, NIBAN2, ECM1, RFX2, SLC40A1, TGFB1, RHOA, TGM2, PPP3R1, MMRN2, SMARCD3, LPAR2, TNFAIP2, PEAK1, PLEKHO1, CDH5, PRRC1
		GO:0034097 (response to cytokine)	3.12E-03	RPLP0, SHFL, RPS2, AXL, GATA3, TCIRG1, RPS16, ADAR, ZYX, LAPTM5, SERPINA1, CXCL9, COL1A1, COL3A1, FN1, SOX17, CASP4, OAS1, PARP14, ECM1, RFX2, IFIT1, RHOA, PIAS4, ISG15, LSP1, ARHGEF2, IL3RA, MX1, HDGF

		GO:0009607 (response to biotic stimulus)	3.12E-03	CYBB, CFHR1, SHFL, AXL, ACTA2, GATA3, MECOM, RPS19, ADAR, ADARB1, ZYX, LAPTM5, H2AC6, H2BC4, FCGR3A, SPON2, CXCL9, C1QB, IFI44L, MMP7, FLOT2, WIPF1, DDX60, GPX1, SIGLEC16, H2AC19, CASP4, RNF213, OAS1, NPC2, PARP14, IFIT1, TGFB1, RHOA, PIAS4, ISG15, C15orf48, MMRN2, TMSB10, INAVA, ARHGEF2, MX1, RPL11, SMAD6
		GO:0051707 (response to other organism)	3.32E-03	CYBB, CFHR1, SHFL, AXL, ACTA2, GATA3, MECOM, RPS19, ADAR, ADARB1, ZYX, H2AC6, H2BC4, FCGR3A, SPON2, CXCL9, C1QB, IFI44L, MMP7, FLOT2, WIPF1, DDX60, GPX1, SIGLEC16, H2AC19, CASP4, RNF213, OAS1, NPC2, PARP14, IFIT1, TGFB1, RHOA, PIAS4, ISG15, C15orf48, MMRN2, TMSB10, INAVA, ARHGEF2, MX1, RPL11, SMAD6
		GO:0043207 (response to external biotic stimulus)	3.32E-03	CYBB, CFHR1, SHFL, AXL, ACTA2, GATA3, MECOM, RPS19, ADAR, ADARB1, ZYX, H2AC6, H2BC4, FCGR3A, SPON2, CXCL9, C1QB, IFI44L, MMP7, FLOT2, WIPF1, DDX60, GPX1, SIGLEC16, H2AC19, CASP4, RNF213, OAS1, NPC2, PARP14, IFIT1, TGFB1, RHOA, PIAS4, ISG15, C15orf48, MMRN2, TMSB10, INAVA, ARHGEF2, MX1, RPL11, SMAD6
		GO:0098542 (defense response to other organism)	3.32E-03	CYBB, CFHR1, SHFL, AXL, GATA3, RPS19, ADAR, ADARB1, ZYX, H2AC6, H2BC4, FCGR3A, SPON2, CXCL9, C1QB, IFI44L, MMP7, FLOT2, DDX60, SIGLEC16, H2AC19, CASP4, RNF213, OAS1, PARP14, IFIT1, TGFB1, PIAS4, ISG15, MMRN2, TMSB10, INAVA, ARHGEF2, MX1, RPL11
		GO:0035295 (tube development)	4.57E-03	CYBB, TNFSF12, ETS1, GATA3, MCAM, MECOM, RPS29, PLEKHG5, ZEB2, MEIS2, ADRB1, ATXN1, RAPGEF3, PDCL3, FKBP8, NOTCH3, COL3A1, COL4A1, HSPG2, TBX3, FBLN5, FN1, WNT11, GPX1, GMFG, SOX17, RNF213, NPC2, NIBAN2, ECM1, TGFB1, RHOA, TGM2, PPP3R1, MMRN2, LPAR2, TNFAIP2, PEAK1, FSCN1, CDH5, SMAD6
		GO:0035455 (response to interferon-alpha)	4.57E-03	AXL, GATA3, ADAR, OAS1, IFIT1
		GO:0071345 (cellular response to cytokine stimulus)	5.18E-03	RPLP0, RPS2, AXL, GATA3, TCIRG1, RPS16, ADAR, ZYX, LAPTM5, CXCL9, COL1A1, FN1, SOX17, CASP4, OAS1, PARP14, ECM1, RFX2, IFIT1, RHOA, PIAS4, ISG15, LSP1, ARHGEF2, IL3RA, MX1, HDGF
		GO:0002181 (cytoplasmic translation)	6.23E-03	RPL27, RPLP0, RPS2, RPS9, RPS16, RPS19, RPS29, RPL23, RPL36, RPL11
		GO:0140236 (translation at presynapse)	6.31E-03	RPL27, RPLP0, RPS16, RPL23, RPL36, RPL11
		GO:0140241 (translation at synapse)	6.31E-03	RPL27, RPLP0, RPS16, RPL23, RPL36, RPL11
		GO:0140242 (translation at postsynapse)	6.31E-03	RPL27, RPLP0, RPS16, RPL23, RPL36, RPL11
		GO:0035457 (cellular response to interferon-alpha)	6.55E-03	AXL, GATA3, OAS1, IFIT1
		GO:0048871 (multicellular organismal-level homeostasis)	7.84E-03	RPL27, AXL, RPS9, ETS1, GATA3, TCIRG1, MECOM, RPS19, RPS29, ADAR, TUB, ZEB2, ADRB1, PIGR, FCGR3A, TPP1, NOTCH3, COL3A1, GPX1, OAS1, SLC40A1, TGFB1, ISG15, SMARCA2, LPAR2, INAVA, CDH5, PRRC1, RPL11
		GO:2000026 (regulation of multicellular organismal development)	7.84E-03	CYBB, ATP5MC1, BTBD6, TNFSF12, AXL, TCIM, ETS1, GATA3, RPS19, ZEB2, MEIS2, S100A10, ADRB1, ATXN1, H4C12, RAPGEF3, FDPS, PDCL3, NOTCH3, HSPG2, TBX3, FBLN5, FN1, WNT11, SOX17, RNF213, NIBAN2, ECM1, TGFB1, RHOA, TGM2, NSMF, ISG15, TM4SF4, SMARCA2, MMRN2, SMARCD3, LPAR2, MAP3K13, ARHGEF2, CDH5
		GO:0034101 (erythrocyte homeostasis)	7.91E-03	RPL27, AXL, RPS9, ETS1, GATA3, RPS19, RPS29, ADAR, SLC40A1, ISG15, LPAR2, RPL11
		GO:0016049 (cell growth)	8.09E-03	ATP5MC1, ZEB2, S100A10, ADRB1, FDPS, LTBP4, FBLN5, FN1, SYT3, WNT11, SOX17, WFDC1, SIPA1, TGFB1, RHOA, POSTN, NSMF, SMARCA2, MAP3K13, IL3RA, CDH5
		GO:0043542 (endothelial cell migration)	8.61E-03	TNFSF12, ETS1, GATA3, PLEKHG5, ZEB2, TAGLN, HSPG2, FN1, GPX1, TGFB1, RHOA, MMRN2, PEAK1, CDH5
		GO:0045087 (innate immune response)	8.61E-03	CYBB, SHFL, AXL, GATA3, RPS19, ADAR, ADARB1, ZYX, H2AC6, H2BC4, FCGR3A, SPON2, C1QB, FLOT2, DDX60, SIGLEC16, H2AC19, CASP4, OAS1, PARP14, IFIT1, TGFB1, PIAS4, ISG15, INAVA, ARHGEF2, MX1, RPL11
		GO:0043009 (chordate embryonic development)	8.74E-03	TPM1, RPL36AL, RPLP0, GATA3, MECOM, RPS19, RPS29, ADAR, ADARB1, ZEB2, MEIS2, SERPINA1, SLC35E2A, FKBP8, NOTCH3, COL1A1, COL3A1, HSPG2, TBX3, NAT8B, FN1, WNT11, SOX17, CASP4, APBA3, TGFB1, CDK2AP1, FSCN1, PRRC1, RPL11
		GO:0097435 (supramolecular fiber organization)	8.74E-03	TPM1, ZEB2, ZYX, TBCB, S100A10, CCDC13, AEBP1, RAPGEF3, TAGLN, LTBP4, COL1A1, COL1A2, COL3A1, HSPG2, NAT8B, FBLN5, WIPF1, WNT11, GPX1, GMFG, CASP4, RHOA, TMSB10, ARHGEF2, FSCN1, CDH5
		GO:0045071 (negative regulation of viral genome replication)	9.14E-03	SHFL, ADAR, OAS1, IFIT1, ISG15, MX1

		GO:0030010 (establishment of cell polarity)	9.47E-03	GATA3, TCIRG1, FLOT2, FN1, WNT11, SIPA1, RHOA, ARHGEF2, FSCN1, CDH5
		GO:0001558 (regulation of cell growth)	9.47E-03	ATP5MC1, S100A10, ADRB1, FDPS, LTBP4, FBLN5, FN1, SYT3, WNT11, SOX17, WFDC1, SIPA1, TGFB1, RHOA, NSMF, SMARCA2, MAP3K13, IL3RA
		GO:0009792 (embryo development ending in birth or egg hatching)	1.03E-02	TPM1, RPL36AL, RPLP0, GATA3, MECOM, RPS19, RPS29, ADAR, ADARB1, ZEB2, MEIS2, SERPINA1, SLC35E2A, FKBP8, NOTCH3, COL1A1, COL3A1, HSPG2, TBX3, NAT8B, FN1, WNT11, SOX17, CASP4, APBA3, TGFB1, CDK2AP1, FSCN1, PRRC1, RPL11
		GO:0002262 (myeloid cell homeostasis)	1.03E-02	RPL27, AXL, RPS9, ETS1, GATA3, MECOM, RPS19, RPS29, ADAR, SLC40A1, ISG15, LPAR2, RPL11
		GO:0034340 (response to type I interferon)	1.10E-02	SHFL, ADAR, OAS1, IFIT1, PIAS4, ISG15, MX1
		GO:0060339 (negative regulation of type I interferon-mediated signaling pathway)	1.10E-02	ADAR, OAS1, PIAS4, ISG15
		GO:0045069 (regulation of viral genome replication)	1.27E-02	SHFL, ADAR, ADARB1, OAS1, IFIT1, ISG15, MX1
		GO:0071229 (cellular response to acid chemical)	1.36E-02	CYBB, ATP5MC1, S100A10, COL1A1, COL1A2, COL3A1, COL4A1, SIPA1, NSMF
		GO:0030218 (erythrocyte differentiation)	1.38E-02	RPL27, RPS9, ETS1, GATA3, RPS19, RPS29, ADAR, SLC40A1, ISG15, LPAR2, RPL11
		GO:0030198 (extracellular matrix organization)	1.44E-02	ETS1, AEBP1, MMP7, LTBP4, COL1A1, COL1A2, COL3A1, COL4A1, HSPG2, FBLN5, FN1, TGFB1, POSTN, FSCN1
		GO:0043062 (extracellular structure organization)	1.44E-02	ETS1, AEBP1, MMP7, LTBP4, COL1A1, COL1A2, COL3A1, COL4A1, HSPG2, FBLN5, FN1, TGFB1, POSTN, FSCN1
		GO:0045229 (external encapsulating structure organization)	1.52E-02	ETS1, AEBP1, MMP7, LTBP4, COL1A1, COL1A2, COL3A1, COL4A1, HSPG2, FBLN5, FN1, TGFB1, POSTN, FSCN1
		GO:0040007 (growth)	1.61E-02	ATP5MC1, AXL, GATA3, ADARB1, ZEB2, S100A10, ADRB1, FDPS, FKBP8, LTBP4, COL1A1, COL3A1, NAT8B, FBLN5, FN1, SYT3, WNT11, GPX1, SOX17, WFDC1, SIPA1, ECM1, TGFB1, RHOA, POSTN, NSMF, WDR11, TM4SF4, SMARCA2, MAP3K13, IL3RA, CDH5
		GO:0001101 (response to acid chemical)	1.93E-02	CYBB, ATP5MC1, S100A10, COL1A1, COL1A2, COL3A1, COL4A1, SIPA1, RHOA, NSMF
		GO:0043534 (blood vessel endothelial cell migration)	2.03E-02	ETS1, PLEKHG5, HSPG2, FN1, GPX1, TGFB1, RHOA, MMRN2, PEAK1, CDH5
		GO:0010935 (regulation of macrophage cytokine production)	2.06E-02	AXL, LAPTM5, SPON2, CASP4, TGFB1
		GO:0001959 (regulation of cytokine-mediated signaling pathway)	2.06E-02	AXL, ADAR, LAPTM5, CASP4, OAS1, PARP14, ECM1, PIAS4, ISG15
		GO:0009617 (response to bacterium)	2.10E-02	AXL, MECOM, RPS19, H2AC6, H2BC4, FCGR3A, SPON2, CXCL9, MMP7, GPX1, SIGLEC16, H2AC19, CASP4, RNF213, OAS1, IFIT1, TGFB1, RHOA, ISG15, C15orf48, MMRN2, TMSB10, INAVA, MX1, SMAD6
		GO:0085029 (extracellular matrix assembly)	2.35E-02	LTBP4, COL1A2, COL3A1, FBLN5, TGFB1
		GO:0048251 (elastic fiber assembly)	2.35E-02	LTBP4, COL3A1, FBLN5
		GO:0060284 (regulation of cell development)	2.35E-02	ATP5MC1, BTBD6, AXL, TCIM, ETS1, GATA3, RPS19, ZEB2, MEIS2, S100A10, ATXN1, H4C12, NOTCH3, FN1, NIBAN2, TGFB1, RHOA, TGM2, NSMF, ISG15, TM4SF4, SMARCA2, SMARCD3, LPAR2, MAP3K13, ARHGEF2, CDH5
	cellular component	GO:0031012 (extracellular matrix)	1.23E-05	ATP5MC1, AXL, ETS1, GATA3, MECOM, ZEB2, ADRB1, ATXN1, FDPS, CXCL9, ADIRF, COL1A1, FN1, SOX17, NIBAN2, TGFB1, MSR1, RHOA, SLC6A6, TGM2, NSMF, ISG15, SMARCA2, SMARCD3, MAP3K13, ARHGEF2, CDH5
		GO:0030312 (external encapsulating structure)	1.23E-05	CYBB, AXL, ETS1, GATA3, TCIRG1, MECOM, RPS19, ZEB2, ZYX, SERPINA1, FCGR3A, CXCL9, HSPG2, FN1, GPX1, GMFG, SIGLEC16, CASP4, WFDC1, NPC2, ECM1, TGFB1, TGM2, TMSB10, CDH5
		GO:0062023 (collagen-containing extracellular matrix)	1.23E-05	ETS1, GATA3, RPS19, ADAR, GPX1, SIGLEC16, OAS1, WFDC1, PARP14, TGFB1, PIAS4, ISG15, CDH5
		GO:0022626 (cytosolic ribosome)	1.23E-05	AXL, ADAR, LAPTM5, CASP4, OAS1, PARP14, ECM1, PIAS4, ISG15

		GO:0005925 (focal adhesion)	2.86E-05	TNFSF12, ACTA2, ETS1, GATA3, PLEKHG5, ZEB2, TAGLN, HSPG2, FN1, GPX1, TGFB1, RHOA, MMRN2, PEAK1, CDH5
		GO:0030055 (cell-substrate junction)	3.60E-05	RHOA, CDH5
		GO:0005581 (collagen trimer)	7.59E-05	RPS9, SLC40A1
		GO:0044391 (ribosomal subunit)	1.16E-04	GATA3, ADARB1, ZEB2, S100A4, COL1A1, HSPG2, TBX3, FN1, WNT11, TGFB1, PPP3R1, WDR11, FSCN1
		GO:0005840 (ribosome)	6.04E-04	CFHR1, AXL, TCIM, ETS1, GATA3, RPS19, ADAR, MEIS2, LAPTM5, PIGR, FCGR3A, H4C12, CD99, SPON2, C1QB, COL3A1, FLOT2, FN1, DDX60, GPX1, SIGLEC16, CASP4, OAS1, PARP14, LCP2, ECM1, TGFB1, RHOA, PIAS4, ISG15, SMARCA2, SMARCD3, LPAR2, INAVA, FYB1, RPL11
		GO:0070161 (anchoring junction)	1.39E-03	AXL, LAPTM5, SPON2, CASP4, TGFB1
		GO:0022625 (cytosolic large ribosomal subunit)	1.83E-03	TNFSF12, ACTA2, ETS1, GATA3, PLEKHG5, ZEB2, TAGLN, HSPG2, FN1, WNT11, GPX1, SOX17, TGFB1, RHOA, WDR11, MMRN2, PEAK1, FSCN1, CDH5
		GO:0015629 (actin cytoskeleton)	1.97E-03	RPL27, AXL, RPS9, ETS1, GATA3, TCIRG1, MECOM, RPS19, RPS29, ADAR, SLC40A1, TGFB1, ISG15, SMARCA2, LPAR2, RPL11
		GO:0098644 (complex of collagen trimers)	2.52E-03	RPS19, ADAR, OAS1, PARP14, TGFB1, PIAS4, ISG15
		GO:0098556 (cytoplasmic side of rough endoplasmic reticulum membrane)	2.83E-03	ACTA2, GATA3, ADARB1, ZEB2, S100A4, NOTCH3, COL1A1, HSPG2, TBX3, FN1, WNT11, TGFB1, PPP3R1, WDR11, FSCN1
		GO:0005584 (collagen type I trimer)	3.63E-03	CYBB, ATP5MC1, TNFSF12, AXL, ETS1, GATA3, MECOM, ZEB2, ADRB1, ATXN1, RAPGEF3, FDPS, PDCL3, CXCL9, ADIRF, COL1A1, FN1, SYT3, WNT11, SOX17, NIBAN2, ECM1, TGFB1, MSR1, RHOA, SLC6A6, TGM2, NSMF, ISG15, SMARCA2, MMRN2, SMARCD3, MAP3K13, ARHGEF2, CDH5
		GO:0030867 (rough endoplasmic reticulum membrane)	3.63E-03	ADAR, OAS1, IFIT1, PIAS4, ISG15, MX1
		GO:0022627 (cytosolic small ribosomal subunit)	5.67E-03	TPM1, S100A10, RAPGEF3, WNT11, RHOA
		GO:0005583 (fibrillar collagen trimer)	6.33E-03	CXCL9, TBX3, FLOT2, TGFB1, SMARCA2, SMARCD3
		GO:0098643 (banded collagen fibril)	6.33E-03	TCIRG1, ADAR, H4C12, MRC2, CHRDL1, COL1A1, COL1A2, HSPG2, FN1, WNT11, ECM1, SLC40A1, TGFB1, RHOA, ISG15, SMAD6
		GO:0015934 (large ribosomal subunit)	7.65E-03	ADAR, MEIS2, CHRDL1, TBX3, FN1, PRRC1, SMAD6
		GO:0098554 (cytoplasmic side of endoplasmic reticulum membrane)	9.62E-03	CFHR1, GATA3, RPS19, ADAR, LAPTM5, PIGR, FCGR3A, C1QB, COL3A1, FLOT2, DDX60, GPX1, SIGLEC16, CASP4, OAS1, PARP14, LCP2, ECM1, TGFB1, PIAS4, ISG15, INAVA, FYB1, RPL11
		GO:0042788 (polysomal ribosome)	1.16E-02	TPM1, ZEB2, ZYX, S100A10, RAPGEF3, WNT11, RHOA
		GO:0043202 (lysosomal lumen)	2.17E-02	TPM1, ZEB2, ZYX, S100A10, RAPGEF3, WNT11, RHOA
		GO:0098794 (postsynapse)	2.81E-02	SHFL, AXL, ADAR, ADARB1, OAS1, IFIT1, ISG15, MX1
		GO:0015935 (small ribosomal subunit)	4.61E-02	ADAR, OAS1, PARP14, ECM1, PIAS4, ISG15
disease controls (41)	molecular function	GO:0002020 (protease binding)	0.0451	MAGEA4, HSPG2, FN1, SERPINB9, NFRKB, TNFAIP3, COL1A1, COL1A2
	biological process	N/A		
	cellular component	GO:0005584 (collagen type I trimer)	0.0228	COL1A1, COL1A2
all controls (48)	molecular function	N/A		
	biological process	N/A		
	cellular component	GO:0005584 (collagen type I trimer)	0.0241	COL1A1, COL1A2
